# Identification and Validation of an Immune-Related lncRNA Signature to Facilitate Survival Prediction in Gastric Cancer

**DOI:** 10.3389/fonc.2021.666064

**Published:** 2021-10-25

**Authors:** Ensi Ma, Sen Hou, Yan Wang, Xiao Xu, Zhengxin Wang, Jing Zhao

**Affiliations:** ^1^ Department of General Surgery, Huashan Hospital, Fudan University, Shanghai, China; ^2^ Institute of Organ Transplantation, Fudan University, Shanghai, China; ^3^ Department of Digestive Surgery, Xuchang Central Hospital, Henan, China; ^4^ Central Laboratory, Huashan Hospital, Fudan University, Shanghai, China; ^5^ Cancer Metastasis Institute, Fudan University, Shanghai, China

**Keywords:** lncRNA signature, gastric cancer, cancer immunity, overall survival, immunosuppressive microenvironment

## Abstract

**Background:**

Long noncoding RNAs (lncRNAs) are versatile in functions and can regulate cancer development, including the modulation of cancer immunity. Immune-related lncRNA signatures predicting prognosis have been reported in multiple cancers, but relevant studies in gastric cancer (GC) are still lacking.

**Methods:**

We performed a comprehensive analysis using TCGA and Immport databases and identified an immune-related lncRNA signature by univariate and multivariate Cox regression analysis. qRT-PCR and immunohistochemistry assays were used for further validation. KEGG and GO analysis and ceRNA network establishment were carried out to explore the regulatory functions.

**Results:**

We first identified an immune-related lncRNA signature, which can stratify gastric cancer patients into high- and low-risk subgroups and the high-risk cases frequently suffered from shorter overall survival time. Next, we validated the reliability of the lncRNA signature in an independent 75 gastric cancer samples and demonstrated that the three-year survival rate in high-risk patients was only 30.8% *versus* 66.5% in low-risk counterparts. Functional exploration indicated that the lncRNA signature might participate in multiple cancer-associated processes including cell adhesion and migration, cytokine-receptor interaction and immune evasion. Additionally, we observed that high-risk samples tended to form an immunosuppressive microenvironment, which had more M2-polarized macrophages and Tregs, but fewer CD8 effector T cells within tumors. Moreover, we found that PD-1 and PD-L1 were dramatically upregulated in a subset of high-risk patients with abundant M2 and Treg infiltration, implying these patients may benefit from anti-PD-1 and PD-L1 immunotherapy.

**Conclusions:**

These results showed that the immune-related lncRNA signature had a prominent capacity to predict overall survival and the immune status of microenvironment in gastric cancer. Our findings may be useful for the risk-stratification management and provide a valuable clue to identify proper patients potentially benefit from immune checkpoint therapy in gastric cancer.

## Introduction

Gastric cancer (GC) is ranked the fifth most frequently diagnosed cancer worldwide and the third leading cause of cancer-related death (1). According to global cancer statistics, there were more than 1,000,000 new gastric cancer cases in 2018, with an estimated 1 in 12 patients dying from the malignancy (1). Surgical resection and chemotherapy are the common curative approaches for gastric cancer. Immunotherapies such as anti-PD-1/PD-L1 immune checkpoint blockade treatment have been demonstrated to be effective in some patients with advanced gastric cancer (2, 3). But the overall prognosis and survival of gastric cancer remain dismal, and the median survival time of advanced patients is only about 1 year (4). To improve the prognosis and reduce the burden of gastric cancer, it is necessary to develop effective molecular biomarkers that identify high-risk patients to build better clinical management and treatment strategies.

The molecular regulatory mechanisms of cancer are extremely complex (5). Beyond the well-documented coding-genes, a number of non-coding genes also exert crucial roles in cancer development. Long noncoding RNAs (lncRNAs) are the primary class of the non-coding family and have been extensively investigated in a variety of cancers (6, 7). LncRNA refers to the transcripts with a length of more than 200 nucleotides but with limited capacity to encode proteins. LncRNAs exhibit versatile functions to manipulate gene expression at the transcriptional, translational, and post-translational levels through binding to DNA, RNA, and proteins (6). Our previous studies identified some lncRNAs that play important roles in gastric cancer progression (8–12). For example, we found that Linc00152 can stimulate proliferation, epithelial to mesenchymal transition (EMT), migration and invasion (8); lncRNA GAS5 has a tumor suppressive role to induce cell cycle G1 arrest and inhibit malignant proliferation (9); and lncRNA PVT1 can activate STAT3 signaling pathway to trigger angiogenesis and vasculogenic mimicry, thereby promoting tumor growth in gastric cancer (10, 12). Recent studies suggest that lncRNAs also have great impacts on cancer immunity. Lnc-NKILA can sensitize tumor-specific cytotoxic T lymphocytes and Th1 cells to undergo activation-induced cell death, thus leading to cancer immune evasion (13). LncRNA-MM2P has the capacity to induce M2 polarization of macrophages, thereby inducing immunological tolerance and tumorigenesis of osteosarcoma (14). In addition to the multifarious functions, lncRNAs are highly stable in physiological environments and can be easily detected in body fluid and extracellular vesicles, therefore regarding as a promising biomarker for early diagnosis and prognostic evaluation (6). Detection of lncRNA PCA3 in urine was approved by the US Food and Drug Administration (FDA) in 2012 to be used in the early diagnosis of prostate cancer (15). Recent studies have reported the predictive value of lncRNA profiles in cancer prognosis, including for gastric cancer (16–19). However, most lncRNA molecular models have lacked adequate exploration of functional associations and necessary validation in independent cohorts.

In the current study, we first identified and then validated an immune-related lncRNA signature that may serve as an independent risk factor for predicting prognosis and survival time in gastric cancer. Our findings provide a potential strategy to identify high-risk patients, which will facilitate the precise management and seek for proper therapeutic strategies for these patients.

## Materials and Methods

### Gastric Cancer Samples Collection

In total, 75 gastric cancer specimens were collected from Huashan Hospital, Fudan University, from August 2015 to December 2016. None of the patients was carried out radio- or chemo-therapy before surgery. Each of the enrolled patients has written the informed consent. This research procedure was approved by the Human Research Ethics Committee of Huashan Hospital, Fudan University. Basic information of the samples was listed in **Supplementary Table S1**.

### RNA Extraction and Quantitative Real-Time PCR Detection

Total RNA was isolated from gastric cancer tissues with Trizol reagent (Invitrogen, U.S.A.) according to the manufacturer’s instructions. Then, the extracted RNA was reverse-transcribed into cDNAs using a PrimeScript RT reagent Kit with genomic DNA Eraser (Takara Bio, Inc., Japan). qRT-PCR was performed using SYBR ^®^ Premix Ex Taq™ kit in the Applied Biosystems Prism 7500. GAPDH was used as the internal control for normalizing the expression of target genes. The primer sequences were listed as follows:

Lnc-SLC26A11-F: 5’-AAGTACAGCGTGTGTCCCAG-3’;
Lnc-SLC26A11-R: 5’-CAACGTGGAGAGGAGGACAC-3’;
Lnc-CHAF1B-F: 5’-CCTCCGAAGAGGATACGCAC-3’;
Lnc-CHAF1B-R: 5’-CTTGGGAAATCCCTGGTGCT-3’;
Lnc-PTPA-3-F: 5’-ACTTGCGGCTGACAAGAGAA-3’;
Lnc-PTPA-3-R: 5’- AATCTGTTCCCTGCTGGGTG-3’;
GAPDH-F: 5’-TCGACAGTCAGCCGCATCTTCTTT-3’;
GAPDH-R: 5’-ACCAAATCCGTTGACTCCGACCTT-3’;

### Immunohistochemistry Assay

For IHC staining, paraffin-embedded tissue slides were deparaffinized by xylene and hydrated in an ethanol gradient. Then, antigen retrieval was carried out in citrate buffer (pH 6.0) at high-temperature. After blocking with 10% normal goat serum, the tissue sections were incubated respectively with a primary CD163, FoxP3 and CD8 antibodies (1:400, Abcam, USA) overnight at 4°C. Subsequently the slides were treated with the ChemMate Dako EnVision Detection Kit, Peroxidase/DAB, Rabbit/Mouse kit (DakoCytomation, Glostrup, Denmark) and counterstained with hematoxylin. The immunostaining signals were observed using Nikon microscope.

### LncRNA Expression Data Collection of Gastric Cancer

Raw gene expression date (HTsq-FPKM) of gastric cancer and the corresponding clinical information were extracted from TCGA database (https://portal.gdc.cancer.gov/) update to May 18, 2020.Transcript data and clinical information of 373 patients were included.

The exclusion criteria were as follows: (i) histologic diagnosis ruled out gastric cancer; (ii) extremely low gene expression values; (iii) Patients with incomplete clinical data and follow-up time less than 30 days. At last, 305 gastric cancer patients with reliable transcript data and detailed clinical information were collected in this research. ID conversion and the classification of RNAs were conducted by Perl (The Perl Programming Language, version 5.30.1, http://www.perl.org). The lncRNAs expression data were selected for further investigation.

### Construction of the Immune-Related lncRNA Signature in Gastric Cancer

The immune-related genes expression profile was downloaded from the Immport database (https://www.immport.org/). Then, co-expression was carried out between the selected gastric cancer lncRNAs and immune-related genes, thereby obtaining the candidate immune-related lncRNA subset in gastric cancer. Next, the relationship of each lncRNA expression with overall survival was calculated using the univariate Cox model, and *P* < 0.01 was considered to be significantly statistical difference. The model was selected and constructed using the internal cohort by backward Cox analysis using Akaike’s information criterion (AIC) selection criteria, where the best model was selected with the least AIC score. The Kaplan‐Meier survival curves, Scatter plots and correlation heatmaps were performed in R software for further analysis (The R Project for Statistical Computing, version3.6.3, https://www.r-project.org/). Finally, lnc-SLC26A11, lnc-CHAF1B and lnc-PTPA-3 were picked out for lncRNA-signature because of their best performance to predict survival in gastric cancer.

### Principal Components Analysis

PCA is a dimension reduction method and has been extensively used in gene expression analysis. More importantly, PCA can remove noise and discover patterns of inherent data through dimensionality reduction. PCA was applied to obtain a low-dimensional cluster distribution of high-dimensional gene sets in this study.

### The Gene Set Enrichment Analysis

GSEA software (version 4.0.3) was employed to predict the potential functions of the lncRNA-signature. Combined with the high- and low-risk patients determined by the risk score system, Gene Ontology (GO) and Kyoto Encyclopedia of Genes and Genomes (KEGG) pathway enrichment analysis were conducted to visualize various genes involved in different pathways and biological functions and their expression patterns. Gene sets for KEGG and GO were downloaded from the Molecular Signatures Database v7.1. Data were corrected for multiple testing (number of permutations=1000).

### The Competing Endogenous RNA Network Development

The ceRNA network of lncRNA-miRNA-mRNA was constructed as follows. First, the potential lncRNA-miRNA interactions were predicted by the DIANA tools (http://carolina.imis.athena-innovation.gr/). Second, miRNAs and their target mRNAs were predicted by searching miRDB (http://mirdb.org/), miRTarBase (http://mirtarbase.mbc.nctu.edu.tw/) and TargetScan (www.targetscan.org) database. Finally, Cytoscape software (version 3.7.2) was employed to visualize the lncRNA-miRNA-mRNA ceRNA network.

### Immunocyte Infiltration Analysis

ESTIMATE (Estimation of Immune cells in MAlignant Tumor tissues using Expression data) was used to infer tumor cellularity as well as the different infiltrating normal cells (20). By performing single-sample gene set-enrichment analysis (ssGSEA), the immune score of each gastric cancer patient was calculated. According to the immune score, the degree of immune cell infiltration was quantified in gastric cancer tissues.

### Statistical Analysis

Kaplan-Meier analysis with log-rank test was carried out to compare the survival difference between high- and low-risk gastric cancer groups. The univariate and multivariate Cox proportional hazard regression analysis were used to evaluate the prognostic value of the immune-related lncRNA signature. The immune scores between high-risk group and low-risk group using Mann-Whitney U test. All the statistical analyses were conducted using the R statistical software.

## Results

### Identification of the Immune-Related lncRNA Signature in Gastric Cancer

**Figure 1:**
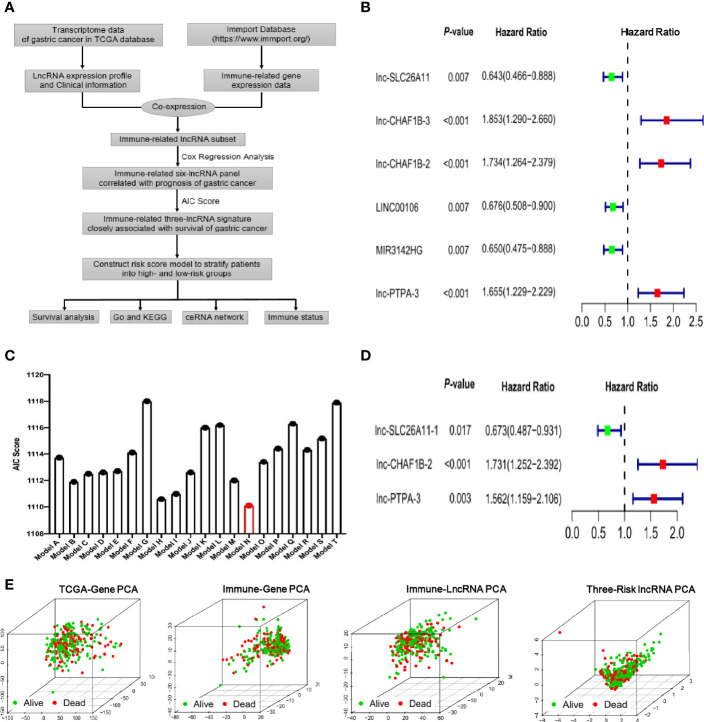
Identification of the immune-related lncRNA signature in gastric cancer. **(A)** Flowchart of identifying immune-related lncRNA signature derived from TCGA transcriptome of gastric cancer and Immport database. **(B)** Forest plot of the six lncRNAs by univariate cox regression analysis. **(C)** Akaike’s information criterion (AIC) scores of different lncRNA combination models and Model N is the best lncRNA signature with the lowest AIC score. **(D)** The multivariate analysis forest plot of Model N, which is composed of three immune-related lncRNAs as indicated in the figure. **(E)** Principal components analysis (PCA) showed survival status among four different gene sets including all genes of TCGA, immune genes of Immport database, immune-related lncRNAs and the identified three-risk lncRNA signature as indicated in the figures.

The Cancer Genome Atlas (TCGA) database across 33 cancer types with detailed clinical information is regarded as a landmark of cancer genomics programs. To identify the immune-related lncRNA signature in the current study, we downloaded all of the raw gene expression data of 373 gastric cancer patients from TCGA database. After carefully checking the clinical information of each sample, 68 patients were excluded due to incomplete clinical data or follow-up time less than 30 days. Ultimately, 305 patients were enrolled in the study and the basic information of gastric cancer patients was listed in **Supplementary Table S1**. The transcriptome data of the enrolled patients was analyzed and the lncRNA expression profile of gastric cancer was extracted from TCGA database. Meanwhile, the immune-gene profile was also acquired from the ImmPort database. By co-expression analysis between the lncRNA profile and immune-gene panel, an immune-lncRNA subset was developed, as depicted in the flowchart of **Figure 1A**. With the help of univariate cox regression analysis, we found that six lncRNAs, lnc-SLC26A11, lnc-CHAF1B-3, lnc-CHAF1B-2, LINC00106, MIR3142HG and lnc-PTPA-3 had significant correlation with prognosis in gastric cancer (**Figure 1B**).

To further optimize the immune-related lncRNA model, Akaike’s information criterion (AIC) was employed to estimate the quality of various lncRNA combination models (**Figure 1C** and **Supplementary Table S2**). As lower AIC score indicate better statistical model quality for a given set of data, the model N with the smallest AIC score was ultimately selected, which was composed of lnc-SLC26A11, lnc-CHAF1B-2 and lnc-PTPA-3. Further multivariate analysis indicated this three-lncRNA model had robust statistical effect to predict prognosis (lnc-SLC26A11: HR = 0.673, 95% CI = 0.487-0.931, *P* = 0.017, coefficient = -0.395; lnc-CHAF1B-2: HR = 1.731, 95% CI = 1.252-2.392, *P* = 0.0009, coefficient = 0.549; lnc-PTPA-3: HR = 1.562, 95% CI = 1.159-2.106, *P* = 0.0034, coefficient = 0.446), as shown in **Figure 1D**.

Principal components analysis (PCA) is a common statistical method used to analyze large gene expression datasets and provide a low dimensional global map to clearly visualize the overall structure of the data. Here, we carried out PCA to evaluate the correlation of four different gene datasets and survival status. As shown in **Figure 1E**, the TCGA-gene dataset, immune-gene from ImmPort database and immune-lncRNA profile nearly failed to divide patients into different subgroups, and the alive and dead cases were unregularly scattered. By comparison, the three immune-related risk lncRNAs could better gather the alive and dead subgroups.

Taken together, the immune-related lncRNA signature was established with lnc-SLC26A11, lnc-CHAF1B and lnc-PTPA attributing to its prominent prognosis predictive potential in gastric cancer.

### Clinical Significance of the Immune-Related lncRNAs in Gastric Cancer

According to the immune-related three-lncRNA signature, a risk score was calculated by regression coefficient for each patient. The risk score calculation was as follows: Risk score = (-0.395* lnc-SLC26A11 expression level) + (0.549*lnc-CHAF1B-2 expression level) + (0.446* lnc-PTPA-3 expression level). The 305 gastric cancer patients were then further divided into high- and low-risk groups based on the median risk score.

**Figure 2:**
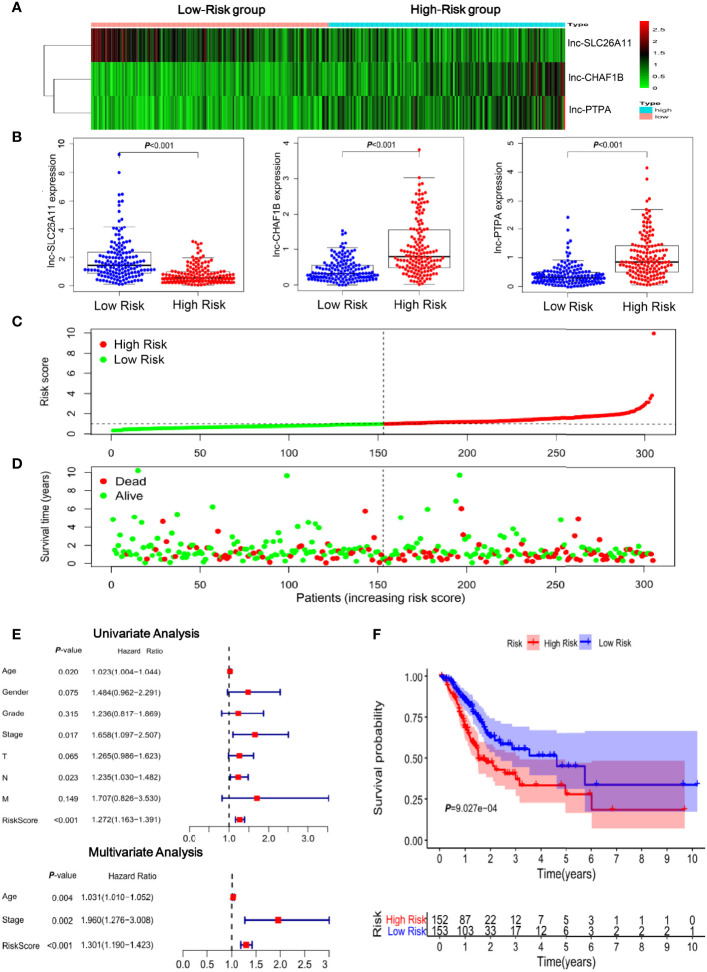
Clinical significance of the immune-related lncRNAs in gastric cancer. **(A)** Heatmap of the three-lncRNA panel. Each column represented a patient. **(B)** The expression levels of the three lncRNAs in gastric cancer samples in TCGA database. **(C)** Risk score distribution according to the immune-related lncRNA profile in TCGA gastric cancer samples. **(D)** The association of risk score and survival status. **(E)** Forest plot of the univariate and multivariate analysis results showed that the risk score could serve as an independent prognostic indicator. **(F)** Kaplan-Meier curves of the overall survival time for high- and low risk patients in TCGA gastric cancer samples.

To clearly visualize the lncRNA signature, the lncRNA expression levels of each patient were displayed in a heat map (**Figure 2A**). Lnc-SLC26A11 was downregulated in high-risk patients, whereas CHAF1B-2 and lnc-PTPA-3 were significantly upregulated in the high-risk group (**Figures 2A, B**). As shown in **Figures 2C, D**, the risk of death gradually increased in accordance with the risk score and there were more dead patients among those with a high-risk score. The univariate and multivariate regression analysis suggested that the risk score could serve as an independent prognostic indicator without linking to age, gender, differentiation grade and clinical stage (**Figure 2E** and **Supplementary Table S3**). Next, we performed Kaplan-Meier analysis to evaluate overall survival between the high-risk and low-risk groups. As shown in **Figure 2F**, patients in the high-risk group frequently suffered from shorter survival time (*P* = 9.027E-04). The 1-year, 3-year, and 5-year survival rate of high-risk compared to low-risk patients were 69.4% *vs*. 84.8%, 40.4% *vs*. 55.4%, and 27.8% *vs*. 45.0%, respectively.

Collectively, these findings suggested that the immune-related lncRNA signature has a great capacity to predict survival of patients with gastric cancer.

### Validation of the Immune-Related lncRNA Signature in an Independent Gastric Cancer Cohort

**Figure 3:**
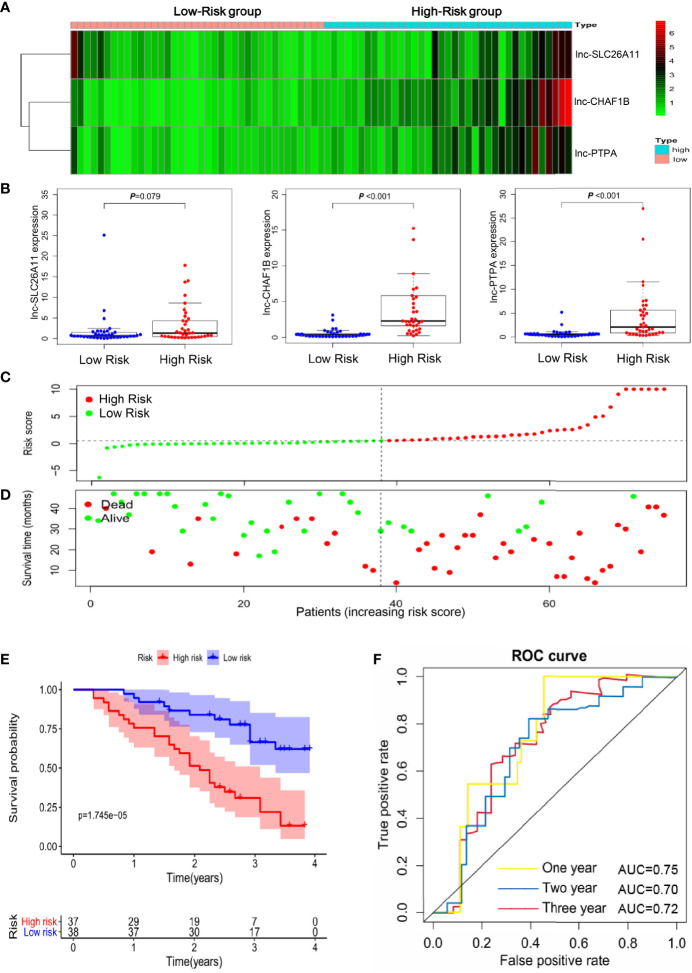
Validation of the immune-related lncRNA signature in an independent gastric cancer cohort. **(A)** Heatmap of the three-lncRNA panel in 75 validated gastric cancer samples. **(B)** The expression levels of the three lncRNAs in the 75 gastric cancer samples. **(C)** Risk score distribution according to the immune-related lncRNA profile in the validated gastric cancer cohort. **(D)** The association of risk score and survival status in the 75 gastric cancer samples. **(E)** Kaplan-Meier curves of the overall survival time for high- and low risk patients in the validated gastric cancer cohort. **(F)** Receiver operating characteristic (ROC) curve analysis showed the area under the receiver-operating curve (AUC) in 1-year, 2-year and 3-year respectively.

To evaluate the reliability of the immune-related lncRNA signature in prognostic prediction, 75 gastric cancer specimens were collected for further validation. The expression levels of the three lncRNAs were determined by qRT-PCR in the specimens and the risk score was calculated for each patient. These gastric cancer patients were stratified into high- and low-risk groups, according to the median risk score. Lnc-SLC26A11 had no statistically significant expression and lnc-CHAF1B-2 and lnc-PTPA-3 expression were markedly increased among the high-risk patients (**Figures 3A, B**). In line with the findings in Fig2, the high-scored patients had higher death risk (**Figures 3C, D**). Additionally, high-risk patients might undergo remarkably shorter survival time, and the 1-year, 2-year, and 3-year survival rates of the high-risk patients compared with those of the low-risk patients were 75.7% *vs*. 94.7%, 51.4% *vs*. 83.9%, and 30.8% *vs*. 66.5%, respectively. (**Figure 3E**). Receiver operating characteristic (ROC) curve analysis showed the immune-related lncRNA signature could serve as a useful prognostic marker for the survival of gastric cancer patients, as the area under the receiver-operating curve (AUC) reached to 0.75, 0.70 and 0.72 in 1-year, 2-year and 3-year respectively (**Figure 3F**).

These results further confirmed the reliability and robustness of the immune-related lncRNA signature in predicting survival of gastric cancer patients, which may help to identify the high-risk patients and build better clinical management at the early stage.

### Potential Functions of the Immune-Related lncRNA Signature

**Figure 4:**
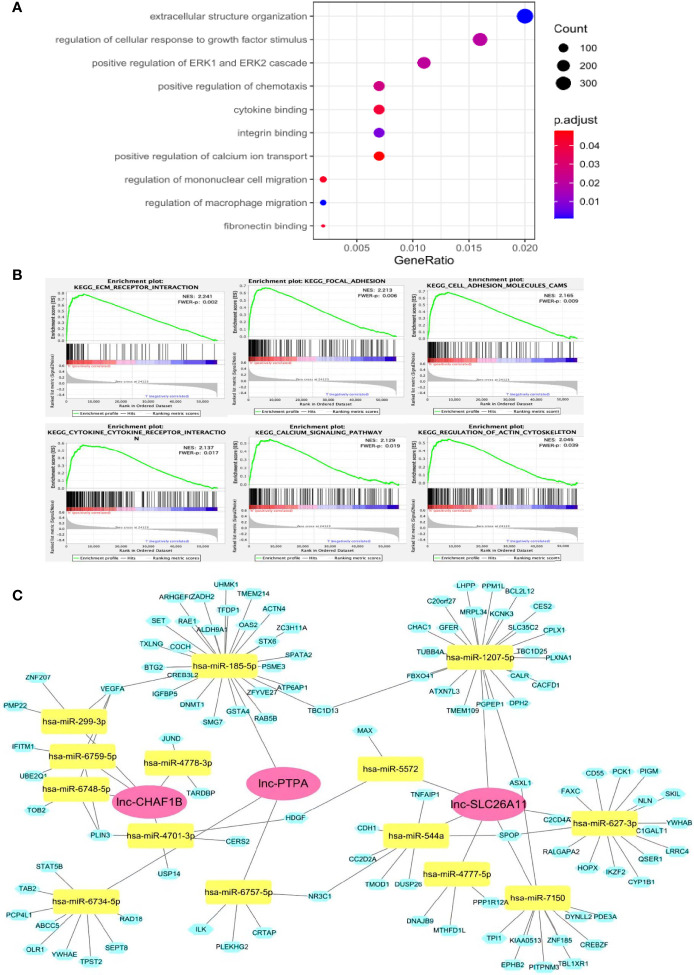
Potential functions of the immune-related lncRNA signature. **(A)** GO analysis showed that these lncRNAs might participate in many cancer development processes. **(B)** GSEA assay indicated that a number of genes were enriched in cancer progression and immune-associated pathways. **(C)** ceRNA network of the lncRNA-signature. The red diamond blocks indicated the lncRNA, the green triangle blocks indicated miRNAs that can be potentially sponged by the lncRNAs. The blue circle blocks indicated the potential mRNA targets of each miRNA.

The functions of lncRNAs are thought to be reflected by their associated protein-coding genes. To exploit the underlying functions of the lncRNA signature, gene ontology (GO) and Kyoto Encyclopedia of Genes and Genomes (KEGG) enrichment analyses were carried out for differentially co-expressed mRNAs. Results of the GO analysis are shown in **Figure 4A**, many cancer-associated processes were identified in the high-risk group by GO analysis, including cellular structure organization, cellular response to growth factor, ERK1/2 cascade, chemotaxis, integrin, cytokine and fibronectin binding and macrophage migration. Cellular structure organization and integrin and fibronectin binding play important roles in cancer metastasis. Meanwhile, cellular response to growth factor stimulus can activate malignant tumor growth, and ERK1/2 cascade is a well-known oncogenic pathway. Macrophage migration, aberrant chemotaxis and cytokine binding may mediate intricate cross-talks between tumors and the tumor microenvironment.

KEGG pathway analysis demonstrated that a number of genes were enriched in cancer progression and immune-associated pathways in the high-risk subset of patients. As shown in **Figure 4B**, ECM receptor interaction, focal adhesion, cell adhesion and actin cytoskeleton regulator are significantly linked to metastasis, whereas cytokine and its receptor interaction, calcium signaling may regulate cancer immunity.

As lncRNA may function as competing endogenous RNA (ceRNA) to sponge miRNA, thereby alleviating the silence effects of miRNA on its mRNA targets, we constructed a lncRNA-miRNA-mRNA ceRNA network to further explore the regulatory mechanisms of our lncRNA-signature (**Figure 4C**). The ceRNA network consisted of 3 lncRNAs, 14 miRNAs and 107 mRNAs. Many of the miRNAs and mRNAs have been previously found to play important roles in different types of cancer development, such as miR-544/RUNX3/NCR1/NKp46 axis in promoting hepatocellular carcinoma development (21), DNMT1 for aberrant DNA methylation (22), VEGFA for cancer-associated angiogenesis (23), among others. The ceRNA network was highly consistent with the GO and KEGG analyses, suggesting our model was convincible and reliable.

Collectively, these results indicated the high-risk group frequently had significant gene enrichment leading to cancer development and stimulating cancer immunity. This provides valuable insight for understanding the functional roles of the newly identified lncRNA signature.

### Correlation of the Three-lncRNA Signature With Infiltration of Immune Cells

**Figure 5:**
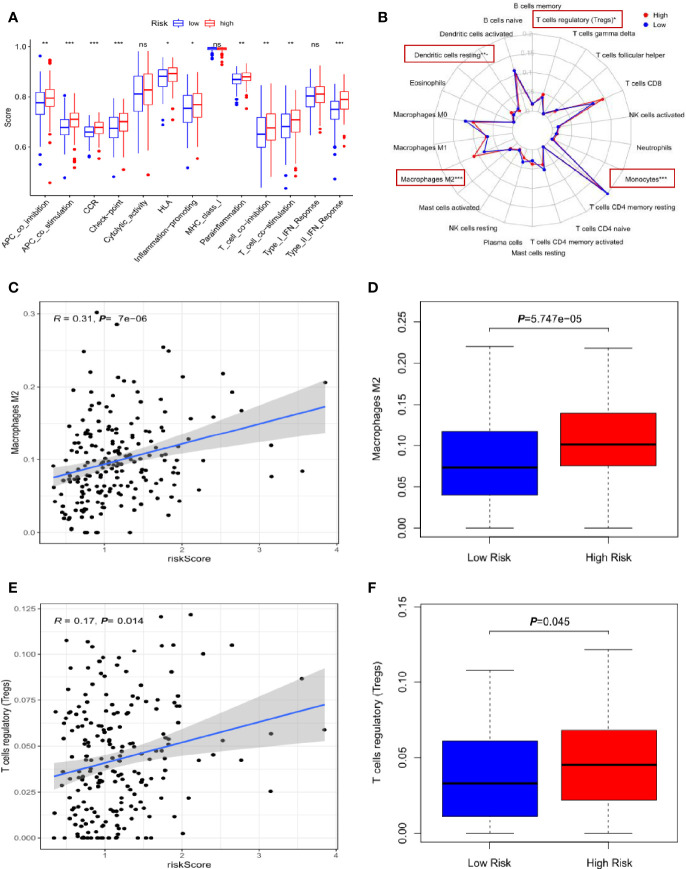
Correlation of the three-lncRNA signature with infiltration of immune cells. **(A)** The comparison of immune-related functions between high- and low-risk groups. **(B)** The radar map showed the infiltration of 22 types of immune cells and the red rectangle boxes indicated the significant difference. **(C)** The scatter plot of M2 polarized cells linked to risk score. **(D)** The relative percentage of M2 polarized cells in high- and low-risk groups. **(E)** The scatter plot of Treg cells infiltration associated with risk score. **(F)** The relative percentage of Treg cells in high- and low-risk groups. *p < 0.05, **p < 0.01, ***p < 0.001. NS means non-statistical significance.

To address the relationship of the lncRNA-signature with cancer immunity, we analyzed some immune-related functions and found that there were significantly functional dysregulation on antigen presenting cell activation, T-cell activation, immune check-point expression, inflammation and interferon responses in high-risk group (**Figure 5A**). Next, the infiltration of 22 types of immune cells were evaluated in low- and high-risk groups, and the red rectangular box showed the differently infiltrated immune cells including macrophage M2, T cells regulatory (Treg), dendritic cell resting and monocytes (**Figure 5B**). It has been widely established that M2 polarized macrophages and Treg cells play critical roles in inducing immune suppression and evasion and aggravate cancer development. Further analysis substantiated that high-risk group had more infiltrating M2 polarized cells compared to that of low-risk group (**Figures 5C, D**). Consistently, Treg cells were enriched in the high-risk specimens (**Figures 5E, F**).

These results implied that the three-lncRNA signature was closely associated with the infiltration of immunosuppressive cells in gastric cancer and high-risk cancer specimens tended to form immunosuppressive microenvironment.

### Immune-Related Gene Expression and Validation in Gastric Cancer Specimens

**Figure 6:**
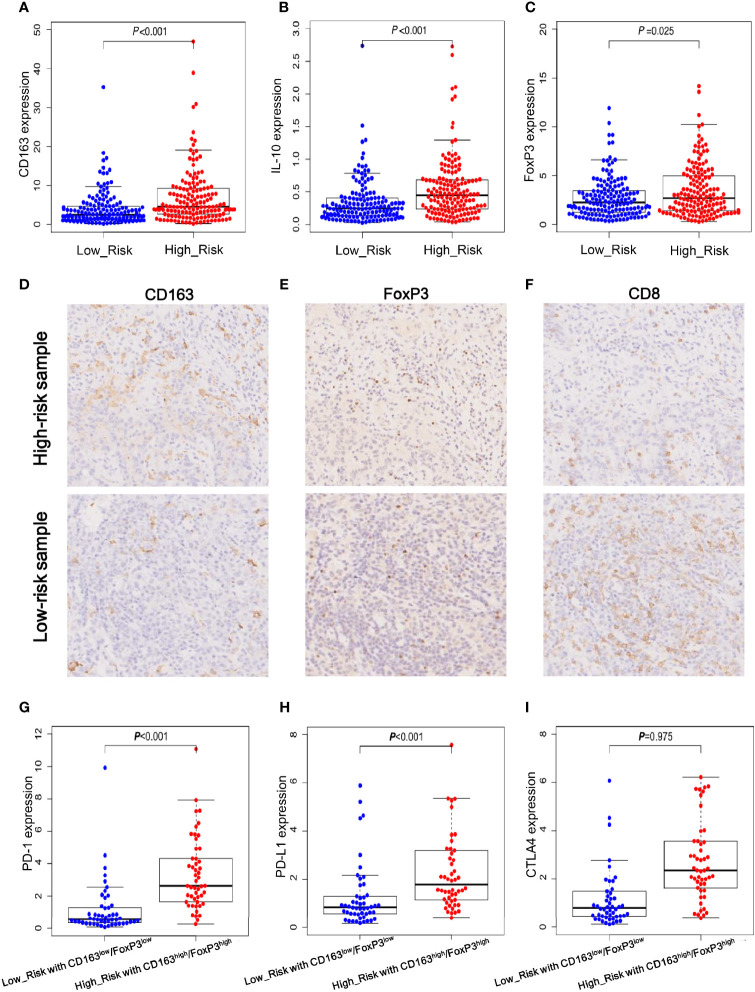
Immune-related gene expression and validation in gastric cancer specimens. **(A, B)** The mRNA expression levels of CD163 and IL-10 which are the well-documented markers of M2 polarized cells. **(C)** The mRNA expression levels of FoxP3 that is a critical for Treg function. **(D–F)** Immunohistochemistry (IHC) staining results of CD163, FoxP3 and CD8 proteins in high- and low-risk gastric cancer samples. **(G–I)** The mRNA expression levels of immune check-point PD-1, PD-L1 and CTLA4 in low-risk group with low expression levels of CD163 and FoxP3 and in high-risk group with high expression levels of CD163 and FoxP3.

It is well-established that CD163 and IL-10 are the markers of M2 polarized macrophages, and FoxP3 is a vital transcriptional factor that mediates Treg function. Thus, the expression levels of these immune-related genes were analyzed in the TCGA dataset and our gastric cancer specimens. As shown in **Figures 6A–C**, the high-risk group exhibited significant upregulation of CD163, IL-10 and FoxP3 transcripts in TCGA dataset. To validate these findings, we carried out immunohistochemistry (IHC) to detect CD163 and FoxP3 proteins in our gastric cancer specimens. Consistent with the mRNA expression results, CD163 and FoxP3 protein levels were remarkably increased in the high-risk samples (**Figures 6D, E**). Meanwhile, the high-risk specimens had less cytotoxic CD8^+^ T-cell infiltration compared to that in the low-risk specimens (**Figure 6F**). The immunosuppressive microenvironment may protect tumor cells from immune destruction, which may account for the poor prognosis in high-risk patients.

Next, according to the expression levels of M2-macrophage and Treg markers, we defined two subgroups for further comparison as follows: the high-risk group with high expression levels of CD163 and FoxP3 and the low-risk group with low expression levels of CD163 and FoxP3. Considering immune checkpoints play vital roles in inhibiting the killing activity of effector T cells and immunotherapies that target immune checkpoints PD-1, PD-L1 and CTLA4 have achieved impressive success in various cancers, we further analyzed the expression levels of the three immune checkpoint molecules in the high- and low-risk subgroups. As shown in **Figures 6G–I**, both PD-1 and PD-L1 were significantly upregulated in high-risk subgroup with high levels of CD163 and FoxP3, and CTLA4 had a trend of increased expression but failed to reach statistical significance. This result imply that immune check-point inhibitors target PD-1/PD-L1 may provide a potential survival advantage for the specific high-risk patients.

Taken together, these findings suggested that the three lncRNAs of signature may have great impacts on the expression of cancer immune-related genes and may modulate the killing activity of CD8^+^ T-cells. Additionally, these results provided some useful clues for the treatment of high-risk patients with enriched infiltration of M2-macrophages and Tregs, who may benefit from anti-PD-1/PD-L1 immunotherapy.

## Discussion

Gastric cancer remains a heavy health burden worldwide due to its high global morbidity and mortality (1). There remains a lack of effective approaches for early diagnosis and prognostic prediction. A large-scale genomic analysis revealed that specific molecular profiles are markedly associated with prognosis of gastric cancer patients (24). Gastric cancer has highly heterogeneous characteristics. In addition to the well-known coding genes and miRNAs, accumulating evidence demonstrates that lncRNAs play crucial roles in gastric cancer development and have great potential to serve as a promising biomarkers and therapeutic targets (6, 15). In the current study, we collected gene expression date and clinical information from TCGA database regarding 305 patients with gastric cancer. We first identified and then validated an immune-related lncRNA signature that prominently correlated with overall survival and immune suppressive status of the patients with gastric cancer. Our findings provide a novel set of lncRNA-based candidates to predict survival and stratify high-risk gastric cancer patients for better promoting early clinical management and treatment.

Given the regulatory functions of lncRNA in cancer immunity, immune-related lncRNA signatures have been exploited in several cancers, including glioblastoma (25), pancreatic cancer (26) and hepatocellular carcinoma (27). These previous investigations demonstrated that the immune-related lncRNA signature has great clinical implication in predicting disease outcomes. However, there have been no studies on immune-related lncRNA signatures in gastric cancer. Although previous studies reported a 24-lncRNA panel and a four-lncRNA combination associated with the prognosis of gastric cancer, these models were limited with respect to specific implications of lncRNA functions and lacked the necessary validation. Here, we identified for the first time an immune-related lncRNA signature of gastric cancer. Our results indicated that the immune-related lncRNA model may serve as an independent risk factor to predict patient survival time. The high-risk patients frequently suffered from shorter survival outcome and exhibited an immune suppressive status. Importantly, these findings were successfully validated using specimens from our independent 75 gastric cancer patients. This further confirmed the reliability and robustness of the immune-related lncRNA model.

To preliminarily understand the functions of the lncRNA signature in gastric cancer, we carried out a series of bioinformatical analyses including GO, KEGG gene set enrichment analyses, evaluating immune-related functions and immune cell infiltration and constructing the ceRNA network. We found that several well-known cancer pathways were enriched by the three-risk-lncRNA panel, including extracellular matrix interaction, cellular adhesion and migration, cytokine-receptor interaction and macrophage migration (**Figure 4**). Notably, high-risk patients showed obvious infiltration of immune suppressive cells including M2 polarized macrophages and Treg cells enrichment (**Figure 5**). IHC assay of our gastric cancer specimens also confirmed that the high-risk samples exhibited stronger M2-macrophage and Treg-related protein expression and had less CD8^+^ T effector cell infiltration (**Figure 6**). M2 polarized macrophages usually shared common characteristics with tumor-associated macrophages (TAMs), which have pleiotropically oncogenic functions to stimulate cancer migration, drug-resistance, immunosuppression and metabolic reprogramming (28). Recent study found that TAM can release extracellular vesicles to package lnc-HISLA and transmit lnc-HISLA to breast cancer cells. TAM-derived lnc-HISLA can stabilize HIF-1α protein to enhance aerobic glycolysis and mediate chemo-resistance of breast cancer cells (29). Treg, as another important category of immunosuppressive cells, can directly prevent cancer cells from antitumor immune responses of cytotoxic T-lymphocyte (30). LncRNA is also found to affect Treg differentiation. For instance, lnc-EGFR can bind to and stabilize EGFR protein to activate AP-1/NFAT signaling axis, which stimulates Treg differentiation and inhibits the cytotoxicity of T cells to promote hepatocellular carcinoma development (31). How the lncRNA signature regulates the infiltration of M2-macrophage and Treg, the underlying mechanisms still need to be clarified in future study. The ceRNA network might provide some implications on the molecular functions of the lncRNA signature. The three risk lncRNAs had the potential to sponge 14 miRNAs and might affect the expression of 107 downstream mRNAs (**Figure 4**). We found that many of the molecules in the network have been demonstrated to aggravate cancer progression. For instance, miR-544 can induce immune escape in hepatocellular carcinoma *via* targeting of RUNX3 to downregulate NCR1/NKp46 (21). Meanwhile, MiR-1207-5p can target STAT6 to promote breast cancer growth (32), DNMT1 is a critical factor responsible for DNA methylation modification (22), VEGFA can trigger aberrant angiogenesis and induce immune evasion (23), and IGFBP5 is capable of regulating the insulin-like pathway (33). These results imply that our immune lncRNA signature may activate cancer-associated signaling pathways, alter epigenetic modification, manipulate immune-related genes expression, affect immune cells recruitment and induce immunosuppressive microenvironment within tumors, which may contribute to poor prognosis and short survival duration in high-risk gastric cancer patients.

In addition, we also attempted to explore potential treatment for the high-risk patients. Nowadays, checkpoint inhibitor-based immunotherapies have made strikingly rapid development to inhibit cancer progression (34). Antibody-mediated blockade of PD-1 and PD-L1 can effectively reinvigorate exhausted T cells and enable them to eradicate tumor cells (35). Anti-PD-1 and PD-L1 antibodies, such as Nivolumab and Pembrolizumab, have displayed a manageable safety and promising efficacy to reduce tumor burden for patients with advanced gastric cancer (2, 3). The positive expression of PD-1 and PD-L1 are commonly used as effective biomarkers to select the responded patients (34). To probe whether high-risk patients may potentially acquire survival advantage from anti-PD-1 and PD-L1 treatment, we compared their transcription levels between high- and low-risk groups but no significant difference was observed (data was not shown). Given tumor immune microenvironment has great impact on the antitumor activity of effector T cells, we stratified a subset of high-risk patients with abundant M2 and Treg infiltration. Inspiringly, we found that both PD-1 and PD-L1 but not CTLA4 were markedly increased in these specific patients (**Figures 6G–I**). This finding implies that the subset of high-risk patients may acquire benefit from the immunotherapy of anti-PD-1 and PD-L1, and thus the lncRNA signature may have the potential to guide treatment option and it is warranted for further investigation.

Despite of the potential clinical implications, this study did have limitations. As the three lncRNAs in our signature have not been annotated previously, there is a lack of experimental evidence on their functional mechanisms. In addition, it will be necessary to evaluate the accuracy and specificity of the lncRNA signature in a prospective study using a larger cohort of gastric cancer samples. Our future investigation will focus on the functional mechanisms of the lncRNA signature and attempt to validate our findings in clinical practice.

In summary, we established an immune-related lncRNA signature in gastric cancer, which showed a prominent potential to predict patient survival and immune-suppressive status. These results provide an alternative strategy for risk-stratification and better clinical management of gastric cancer patients.

## Data Availability Statement

The datasets presented in this study can be found in online repositories. The names of the repository/repositories and accession number(s) can be found in the article/**Supplementary Material**.

## Ethics Statement

The studies involving human participants were reviewed and approved by Human Research Ethics Committee of Huashan Hospital, Fudan University. The patients/participants provided their written informed consent to participate in this study.

## Author Contributions

JZ and ZW conceived of and designed the study. EM and SH collected data, constructed the lncRNA model and collected gastric cancer samples. EM, YW and XX performed the experiments. JZ and ZW analyzed the data and drafted the manuscript. All authors critically revised the work and approved the final manuscript.

## Funding

This study was supported by the Natural Science Foundation of China (No. 82173093, 81672378, 81201521, 81873874 and 81773089) and the National Key Project for Infectious Disease of China (2017ZX10203205-002-004 and 2017ZX10203205-003-003).

## Conflict of Interest

The authors declare that the research was conducted in the absence of any commercial or financial relationships that could be construed as a potential conflict of interest.

The handling editor FL declared a shared parent affiliation with the authors EM, YW, XX, ZW, and JZ at the time of the review.

## Publisher’s Note

All claims expressed in this article are solely those of the authors and do not necessarily represent those of their affiliated organizations, or those of the publisher, the editors and the reviewers. Any product that may be evaluated in this article, or claim that may be made by its manufacturer, is not guaranteed or endorsed by the publisher.
