# Supplementary material for: Identification and Validation of an Immune-Related lncRNA Signature to Facilitate Survival Prediction in Gastric Cancer
Source: Front Oncol. 2021 Oct 25;11:666064. doi: 10.3389/fonc.2021.666064 (PMC8573392; doi:10.3389/fonc.2021.666064)
Supplement: Supplementary file 1 [file DataSheet_1.pdf]

Table S1. The basic information of gastric cancer samples in this study

Characteristics	Our gastric cancer cohort （n=75）	TCGA gastric cancer samples （n=305）
**Age (years)**		
<=60	28	102
>60	47	203
**Gender**		
Male	58	193
Female	17	112
**Tumor size (cm)**		
<=3cm	27	157
>3cm	48	3
**Lymphatic Metastasis**		
Yes	19	207
No	56	89
**TNM Stage**		
I&II	19	139
III&IV	56	153
**Histological Grade**		
I&II	18	117
III&IV	57	181
