# Supplementary material for: Identification and Validation of an Immune-Related lncRNA Signature to Facilitate Survival Prediction in Gastric Cancer
Source: Front Oncol. 2021 Oct 25;11:666064. doi: 10.3389/fonc.2021.666064 (PMC8573392; doi:10.3389/fonc.2021.666064)
Supplement: Supplementary file 2 [file DataSheet_2.pdf]

Table S2 Akaike's information criterion (AIC) score of various lncRNA combination models

Model name	Candidate lncRNA panels	AIC Score
Model A	AC124319.1 + AP000695.1 + AP000695.2 + LINC00106 + MIR3142HG + AL161785.1	1113.73
Model B	AC124319.1 + AP000695.2 + LINC00106 + MIR3142HG + AL161785.1	1111.9
Model C	AC124319.1 + AP000695.1 + AP000695.2 + MIR3142HG + AL161785.1	1112.5
Model D	AC124319.1 + AP000695.1 + LINC00106 + MIR3142HG + AL161785.1	1112.6
Model E	AC124319.1 + AP000695.1 + AP000695.2 + LINC00106 + AL161785.1	1112.7
Model F	AP000695.1 + AP000695.2 + LINC00106 + MIR3142HG + AL161785.1	1114.1
Model G	AC124319.1 + AP000695.1 + AP000695.2 + LINC00106 + MIR3142HG	1118
Model H	AC124319.1 + AP000695.2 + MIR3142HG + AL161785.1	1110.6
Model I	AC124319.1 + AP000695.2 + LINC00106 + AL161785.1	1111
Model J	AP000695.2 + LINC00106 + MIR3142HG + AL161785.1	1112.6
Model K	AC124319.1 + LINC00106 + MIR3142HG + AL161785.1	1116
Model L	AC124319.1 + AP000695.2 + LINC00106 + MIR3142HG	1116.2
Model M	AC124319.1 + AP000695.2 + AL161785.1 + AP000695.1	1112
Model N	AC124319.1 + AP000695.2 + AL161785.1	1110.1
Model O	AP000695.2 + MIR3142HG + AL161785.1	1113.4
Model P	AC124319.1 + AP000695.2 + MIR3142HG	1114.4
Model Q	AC124319.1 + MIR3142HG + AL161785.1	1116.3
Model R	AP000695.2 + AL161785.1	1114.3
Model S	AC124319.1 + AP000695.2	1115.2
Model T	AC124319.1 + AL161785.1	1117.9

The red marked model N had the smallest AIC score which indicates the best statistical model quality for a given set of data.

AC124319.1 is named lnc-SLC26A11, AP000695.2 is named lnc-CHAF1B-2 and AL161785.1 is named lnc-PTPA-3 in LNCipedia database (https://lncipedia.org/db/transcript/)
