# Supplementary material for: Identification and Validation of an Immune-Related lncRNA Signature to Facilitate Survival Prediction in Gastric Cancer
Source: Front Oncol. 2021 Oct 25;11:666064. doi: 10.3389/fonc.2021.666064 (PMC8573392; doi:10.3389/fonc.2021.666064)
Supplement: Supplementary file 3 [file DataSheet_3.pdf]

Table S3 Univariate and multivariate Cox regression analysis of the immune-related lncRNA signature in gastric cancer cohort

Variables	Univariate Cox regression model		Multivariate Cox regression model	
	**HR (95%CI)**	***p* Value**	**HR (95%CI)**	***p* Value**
**Age** (>=60 vs. <60)	1.023 (1.004-1.044)	***0.020***	1.031 (1.010-1.052)	***0.004***
**Gender** (male vs. female)	1.484 (0.962-2.291)	0.075	1.450 (0.925-2.271)	0.105
**TMN stage**				
**Tumor topography** (with vs. without)	1.265 (0.986-1.623)	0.065	1.152 (0.811-1.637)	0.431
**Lymph node** (with vs. without)	1.235 (1.030-1.482)	***0.023***	1.049 (0.798-1.380)	0.730
**Metastasis** (with vs. without)	1.707 (0.826-3.530)	0.149	1.524 (0.602-3.861)	0.374
**Histological grade** (III-IV vs. I-II)	1.236 (0.817-1.869)	0.315	1.189 (0.785-1.802)	0.413
**Tumor stage** (III-IV vs. I-II)	1.658 (1.097-2.507)	***0.017***	1.960 (1.276-3.008)	***0.002***
**Risk Score** (high- vs. low risk)	1.272 (1.1631-1.391)	***1.37E-07***	1.301 (1.190-1.423)	***1.88E-07***
